# MHF1-2/CENP-S-X performs distinct roles in centromere metabolism and genetic recombination

**DOI:** 10.1098/rsob.180010

**Published:** 2018-02-14

**Authors:** Sonali Bhattacharjee, Fekret Osman, Laura Feeney, Alexander Lorenz, Claire Bryer, Matthew C. Whitby

*Open Biol*. **3**, 130102. (Published online 11 September 2013). (doi:10.1098/rsob.130102)

We wish to retract the research paper ‘MHF1-2/CENP-S-X performs distinct roles in centromere metabolism and genetic recombination’. We have discovered that six of the fission yeast strains used in this study (MCW5895, MCW5932, MCW6001, MCW6141, MCW6142 and MCW6152) contain a *fml1*ΔC^1-603^::*natMX4* mutation instead of the stated *fml1*^AAA^::*natMX4* mutation. When we re-analysed strains with the correct *fml1*^AAA^::*natMX4* mutation, we were unable to reproduce these reported observations: an increase in meiotic crossovers (figure 4*d*; electronic supplementary material, table S1); a reduction in *RTS1*-induced direct repeat recombination (figure 5*c*); a change in Mhf1-GFP nuclear localization (figure 6); and an increase in the number and length of mitotic bridges (figure 7*c*,*d*). Moreover, a *fml1*^AAA^::*natMX4* strain exhibits only very modest hypersensitivity to MMS, which is significantly less than shown in figure 3*b*. We have also discovered that a *fml1*ΔC^1-603^::*natMX4* mutant exhibits a similar hypersensitivity to genotoxins as a *fml1*Δ strain, and not the intermediate level of sensitivity shown in figure 3*b*. However, we were able to reproduce the result that a *fml1*^AAA^::*natMX4* mutant exhibits an increase in mitotic crossovers (figure 4*b*) and confirm that a C-terminal fragment of Fml1, containing the AAA mutation, has a weakened interaction with Mhf1–Mhf2 *in vitro* (figure 2*g*). Data from the analysis of the *fml1*^AAA^::*natMX4* mutant were used to support our conclusion that a direct physical interaction between Fml1 and Mhf1–Mhf2 is needed to promote Fml1's activities in DNA repair and recombination. In our recent work, we have been able to validate this conclusion using a mutation that more severely weakens the interaction between Fml1 and Mhf1–Mhf2 than the AAA mutation (Neo J.P.S., Wong I.N., Osman F. and Whitby M.C. 2016, unpublished data). We have also confirmed that the other data in our paper are robust and reproducible and the central conclusions of the paper remain true. We sincerely apologize for any inconvenience that publication of the data pertaining to the *fml1*^AAA^::*natMX4* mutant has caused for others.

Sonali Bhattacharjee

One Bungtown Road, Cold Spring Harbor, NY 11724

bhattacharjee@cshl.edu

Fekret Osman

Laura Feeney

Memorial Sloan Kettering Cancer Center, 430 East 67th Street, New York, NY 10065

feeneyl@mskcc.org

Alexander Lorenz

University of Aberdeen, Institute of Medical Sciences, Aberdeen AB25 2ZD

a.lorenz@abdn.ac.uk

Claire Bryer

Institute of Cancer and Genomic Sciences, College of Medical and Dental Sciences, University of Birmingham, B15 2TT

C.Bryer@bham.ac.uk

Matthew C. Whitby

University of Oxford, South Parks Road, Oxford OX1 3QU

matthew.whitby@bioch.ox.ac.uk

Prof. David M. Glover FRS

Editor-in-Chief, Open Biology

